# Chronic hypoxaemia and gender status modulate adiponectin plasmatic level and its multimer proportion in severe COPD patients: new endotypic presentation?

**DOI:** 10.1186/s12890-020-01288-3

**Published:** 2020-10-01

**Authors:** Mélany Pierard, Alexandra Tassin, Antoine Legrand, Alexandre Legrand

**Affiliations:** 1 Laboratory of Respiratory Physiology, Pathophysiology and Rehabilitation, Research Institute for Health Sciences and Technology, University of Mons, Avenue du Champ de Mars, 6, B-7000 Mons, Belgium; 2 Department of Pneumology, Erasme Hospital, Brussels, Belgium

**Keywords:** COPD, Gender difference, Hypoxaemia, Hypoxia, Adiponectin, Multimers

## Abstract

**Background:**

Disease progression in COPD patient is associated to lung function decline, leading to a higher risk of hypoxaemia and associated comorbidities, notably cardiovascular diseases (CVD). Adiponectin (Ad) is an adipokine with cardio-protective properties. In COPD patients, conflicting results were previously reported regarding Ad plasmatic (Ad_pl_) level, probably because COPD is a heterogeneous disease with multifactorial influence. Among these factors, gender and hypoxaemia could interact in a variety of ways with Ad pathway. Therefore, we postulated that these components could influence Ad_pl_ level and its multimers in COPD patients and contribute to the appearance of a distinct endotype associated to an altered CVD risk.

**Methods:**

One hundred COPD patients were recruited: 61 were men and 39 were women. Patients who were not severely hypoxemic were allocated to non-hypoxemic group which included 46 patients: 27 men and 19 women. Hypoxemic group included 54 patients: 34 men and 20 women. For all patients, Ad_pl_ level and proportion of its different forms were measured. Differences between groups were evaluated by Rank-Sum tests. The relationship between these measures and BMI, blood gas analysis (*P*aO_2_, *P*aCO_2_), or lung function (FEV1, FEV1/FVC, TL_CO_, TLC, RV) were evaluated by Pearson correlation analysis.

**Results:**

Despite similar age, BMI and obstruction severity, women had a higher TLC and RV (median: TLC = 105%; RV = 166%) than men (median: TLC = 87%; RV = 132%). Ad_pl_ level was higher in women (median = 11,152 ng/ml) than in men (median = 10,239 ng/ml) and was negatively associated with hyperinflation (R = − 0,43) and hypercapnia (R = − 0,42). The proportion of the most active forms of Ad (HMW) was increased in hypoxemic women (median = 10%) compared with non-hypoxemic women (median = 8%) but was not modulated in men.

**Conclusion:**

COPD pathophysiology seemed to be different in hypoxemic women and was associated to Ad modulations. Hyperinflation and air-trapping in association with hypercapnia and hypoxaemia, could contribute to a modulation of Ad_pl_ level and of its HMW forms. These results suggest the development of a distinct endotypic presentation, based on gender.

## Background

Chronic obstructive pulmonary disease (COPD) is a progressive disease associated with lung function decline and comorbidities such as cardiovascular diseases (CVD). By inducing secondary erythrocytosis, endothelial dysfunction, and pulmonary arterial hypertension, hypoxaemia in severe COPD patients was suggested to contribute to the increased CVD risk in these patients [1, 2]. The prevalence of the disease is increasing among women due to the higher smoking rate [3, 4]. Moreover, sex-differences were reported in the course of the disease. Women were described to exhibit a greater susceptibility to tobacco, faster annual decline of lung function and worse quality of life [3, 4]. This increased effect of tobacco smoke on women was suggested to be due to gender differences in airway structure [5–7], but inflammatory response to tobacco smoke was also suspected to differ at the level of the small airways [8] and to be at the origin of a more extensive airway remodelling [9]. The prevalence of co-morbidities also varies with gender in COPD patients and men were described to be more susceptible to CVD and diabetes mellitus [10].

Adiponectin (Ad), a 30 kDa protein mainly secreted by adipose tissue, was well described for its anti-inflammatory, anti-atherogenic and anti-diabetic effects [11]. The physiological plasmatic concentration of this adipokine is higher in women [12]. In circulating blood, Ad was detected in 3 different isoforms: low (LMW), medium (MMW), and high molecular weight forms (HMW). This latter form was described as the most active isoform: it was better correlated to insulin sensitivity and circulating glucose concentration [13, 14]. A reduced Ad plasmatic (Ad_pl_) level was associated to multiple metabolic and CVD disorders [15]. In COPD patients, conflicting results regarding Ad_pl_ level were observed in previous studies [16–18] and could be explained by the heterogeneity of the disease. Several factors, such as exacerbation rate, BMI, disease severity and progression were associated to differences in Ad_pl_ level in COPD [16, 19–22]. In addition, exposure to chronic hypoxia was also shown to alter Ad expression in adipocytes in vitro, and in adipose tissue in vivo [23–25].

Adiponectin was previously proposed as a biomarker for COPD risk management, and its pathway was suggested as a potential therapeutic target [20, 26]. However, COPD is a complex and heterogeneous disease [27] that could interact in a variety of ways with Ad pathway [16, 19–22]. In this study, we therefore evaluated Ad_pl_ level and the proportion of HMW forms in COPD patients, with a special attention to gender and hypoxaemia effects. Potential relationships between these data and lung function were also evaluated.

## Methods

### Subjects

One hundred COPD patients were recruited from the outpatient clinic of a tertiary University Hospital from 2016 to 2018 and were referred for evaluation of need for oxygen therapy or for the adaptation of this treatment. COPD was diagnosed according to the ACCP/ATS/ERS guidelines [28] and severity of their airflow limitation was defined according to GOLD criteria, described in [29].

In this study, patients were divided into several groups according to gender and/or the severity of hypoxaemia. Among all patients of the study, 61 were men and 39 women. Concerning the hypoxaemia, patients were affected the hypoxemic group when they were severely hypoxemic patients (PaO_2_ ≤ 55 mmHg) or to the non-hypoxemic group when their PaO_2_ was above 55 mmHg, while breathing room air. This cut-off was based on ATS/ERS statement in which severe hypoxaemic COPD patients were defined as patients exhibiting a PaO_2_ ≤ 55 mmHg. Non-hypoxemic group included 46 patients (27 men and 19 women), whereas hypoxemic group included 54 patients (34 men and 20 women).

Patients with significant comorbidities such as malignancy, endocrine, liver, or gastrointestinal disorders, primitive cardiovascular abnormalities, or a recent surgery were excluded. All subjects were older than 40 years of age. This study was approved by the Erasme Hospital Ethics Committee and conducted in accordance with the Helsinki Declaration.

Patients’ data including age, gender and BMI were collected, and analyses were performed anonymously. Arterial blood samples were obtained at rest, with the patient in the sitting position, while breathing room air. Arterial oxygen (*P*aO_2_) and carbon dioxide (*P*aCO_2_) partial pressures were measured and recorded. Spirometry, lung volumes and single-breath determination of carbon monoxide uptake were then evaluated according to the ATS guidelines [30–32] (M.E.C PFT systems Body™ and Diff™, Belgium) and expressed as a percentage of the predicted value (%pv). FEV1 (forced expiratory volume in one second) and FVC (forced vital capacity), FEV1/FVC ratio, carbon monoxide transfer factor (TL_CO_), total lung capacity (TLC), and residual volume (RV) were recorded. As patients did not interrupt their medical treatment before medical appointment, these measures could be reasonably considered as post-bronchodilation data. TLC and RV have not been obtained from 2 non-hypoxemic women and 5 men because they were unable to perform plethysmography in a reproducible way.

### Adiponectin plasmatic level measurement

Ad_pl_ level was measured according to the manufacturer’s instructions (DRP300: Human Total Adiponectin/Acrp30 Quantikine ELISA Kit, R&D Systems). Assay sensitivity was estimated at 0.891 ng/ml and assay range was between 3.9 and 250 ng/ml. Patient samples were diluted according to the manufacturer’s instructions in order to be within the predefined range.

### Adiponectin oligomer distribution determined by Western blot

The relative amounts of LMW, MMW and HMW Admer were evaluated for each patient as previously described in Pierard et al. [33]. These amounts were determined using a non-denaturing PAGE-SDS followed by a Western blot [33]. Five microliter of plasma diluted to contain 5 μg/ml of Ad were loaded onto a 6% polyacrylamide gel in the presence of SDS [33]. For the Western blot, proteins were transferred to a nitrocellulose membrane (Millipore, Darmstadt, Germany) [33]. After blocking with 5% fat-free dry milk-TBS, the membranes were incubated with a rabbit polyclonal primary antibody directed against Ad (Ab85827, 1:1000, Abcam, Cambridge, UK) [33]. The membranes were then incubated with a horseradish peroxidase-labelled secondary antibody (1:5000, Sigma-Aldrich, St. Louis, MO, USA) [33]. The ECL™ Western Blotting Detection kit (GE Healthcare, Little Chalfont, UK) was used for the revelation step [33]. The immunoreactive bands were then submitted to a densitometric analysis using the Image J software to determine the proportion of LMW, MMW and HMW forms [33].

### Statistical analysis

Sample size was estimated for a 4-group ANOVA study with a power of 0.8 and the significance level at 0.05. The expected difference in means was based on the difference in HMW% observed in mice [34] and the variation among experimental subjects was arbitrarily considered as twice as high as in this mouse study. The total sample size was rounded to 100 patients. A Rank-Sum test was used to evaluate differences between groups. In the tables, results were represented as median and 5th–95th percentiles. In the graphs, all data were represented as boxplots (5th–95th percentiles; dots are outliers). Pearson’s coefficient was used for correlation analyses. Pearson correlation coefficient (R) was calculated between every lung function parameter and Ad_pl_ level measured by ELISA or HMW form proportion determined by non-denaturant SDS-PAGE followed by a Western blot. All representative blots were included in supplementary material 1 and all data obtained to perform these correlations were included in supplementary material 2. Differences were considered statistically significant at a *P* value < 0.05. All statistical analyses were performed with Sigma Plot 11.0 Software (Systat Software, USA).

## Results

### Characteristics of subjects

One hundred COPD patients with moderate (16%), severe (43%) or very severe obstruction (41%) were included in this study. 61% of the cohort were male. 46% were not severely hypoxemic and allocated to the non-hypoxemic group (Table 1). On average, hypoxemic and non-hypoxemic patients exhibited the same age, BMI and disease severity based on FEV1 value. Both groups had a significant air trapping (increased RV). The alteration of TL_CO_ was more severe in hypoxemic patients and these patients were more hypercapnic than non-hypoxemic counterparts. When the cohort was separated according to gender, no difference was observed for age, BMI, FEV1, *P*aCO_2_ or TL_CO_. However, we found an increased TLC and RV in women compared with men.

**Table 1:** Clinical characteristics of COPD subjects separated according to gender or hypoxaemia status. Median _[25th – 75th percentiles]_. * *p* < 0.05; **p < 0,001; Rank-Sum test

	**Non-hypoxemic** (*n* = 46)	**Hypoxemic** (*n* = 54)	***P*** **value**
Age (year)	68 _[65–76]_	71,5 _[64–77]_	0,745
BMI (kg/m^2^)	24,3 _[20,2 – 30,5]_	25,8 _[22,4 – 30,1]_	0,531
*P*aO_2_ (mmHg)	62 _[59–66]_	47 _[41–51]_	< 0,001**
*P*aCO_2_ (mmHg)	41 _[37–44]_	45,5 _[39–53]_	0,002*
*pH*	7,42 _[7,40 – 7,45]_	7,42 _[7,38 – 7,44]_	0,535
FEV_1_ (%pv)	34,5 _[29_–_46]_	31 _[25–44]_	0,186
FEV_1_/FVC	46,8 _[38–58]_	46,3 _[39,5–52]_	0,445
TL_CO_ (%pv)	39,5 _[32,5 – 49,5]_	29,5 _[23–42,8]_	0,002*
TLC (% pv)	91 _[79–104,8]_	97,5 _[82–109,8]_	0,225
RV (%pv)	143 _[117,5–171]_	152, 65 _[114–202,5]_	0,334
Ad_pl_ level (ng/ml)	11,599,1 _[6021,6 – 15,073,0]_	9804,7 _[5857,8 – 13,306,2]_	0,483
	**Men** (*n* = 61)	**Women** (*n* = 39)	
Age (year)	72 _[66,7 – 77,2]_	67 _[60–74,7]_	0,061
BMI (kg/m^2^)	25,6 _[22,4 – 30,5]_	24,8 _[20,3 – 30,6]_	0,799
*P*aO_2_ (mmHg)	54 _[45–60,5]_	55 _[47–61,7]_	0,538
*P*aCO_2_ (mmHg)	42 _[37–47]_	45 _[40–50]_	0,127
*pH*	7,42 _[7,40 – 7,44]_	7,42 _[7,39 – 7,45]_	0,450
FEV_1_ (%pv)	32 _[25,7 – 44,2]_	34 _[27,2–46]_	0,424
FEV_1_/FVC	47 _[39,7 – 55,2]_	45,8 _[39,0 – 54,8]_	0,835
TL_CO_ (%pv)	35 _[26,6 – 50,5]_	32,9 _[24–43,3]_	0,254
TLC (% pv)	87,35 _[74,5 – 102,5]_	105,4 _[90–116]_	< 0,001**
RV (%pv)	132,3 _[104–179]_	166,25 _[138–195]_	0,008*
Ad_pl_ level (ng/ml)	10,239,2 [5265,7-13,062,8]	11,152,232 [7549,7-19,022,8]	0,044*

### Effect of hypoxaemia on Ad_pl_ level and HMW forms

**Fig. 1:**
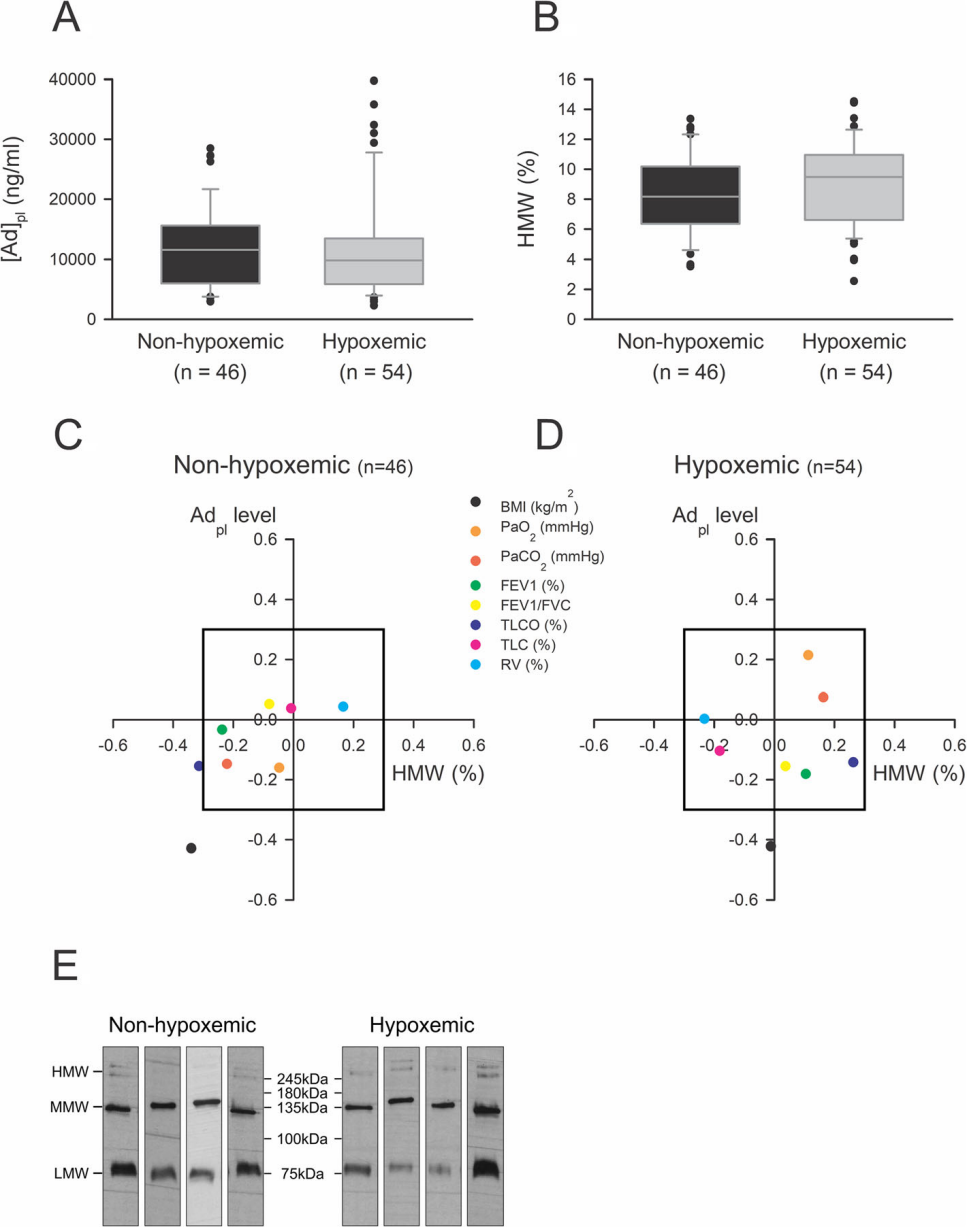
**a-b** Ad_pl_ level (**a**) and HMW form proportion (**b**) in non-hypoxemic and hypoxemic COPD patients. Ad_pl_ level was measured by ELISA and HMW form proportion was determined using a non-denaturing PAGE-SDS followed by a Western blot. Data were represented as boxplots (5th and 95th percentiles). Rank-Sum test: NS. **c**-**d** Correlation between lung function parameters and Ad_pl_ level (vertical) or HMW form proportion (horizontal), in non-hypoxemic (**c**) and hypoxemic (**d**) COPD patients. Pearson correlation coefficients (R) between every parameter and either Ad_pl_ level or HMW form proportion were calculated and represented as a point on the graph. The box in the graph represents the critical value for Pearson correlation coefficient to obtain a *p* < 0.05. Therefore, every point outside the box corresponds to a significant correlation. **e** Representative blots of Ad form proportions in hypoxemic and non-hypoxemic patients, determined by using non-denaturant PAGE-SDS followed by a Western blot. HMW forms correspond to the higher molecular weight bands. Admer/total Ad ratios were obtained after densitometric analysis

We did not observe any difference in Ad_pl_ level and in HMW form proportion between hypoxemic and non-hypoxemic patients (Fig. 1a-b, e). We found that Ad_pl_ level was negatively correlated with BMI in both groups but was not correlated with other parameters (Fig. 1c-d, Table 2). We detected a significant negative correlation between BMI and HMW form proportion in non-hypoxemic patients, but not in the hypoxemic group. Moreover, a negative correlation was observed between HMW forms and TL_CO_ in non-hypoxemic patients. In the hypoxemic group, these parameters were positively correlated but without reaching a statistically significant level (*p* = 0.055).

**Table 2:** Correlation between lung function parameters and Ad_pl_ level or HMW form proportion in non-hypoxemic and hypoxemic COPD patients. Ad_pl_ level was measured by ELISA and HMW form proportion was determined using a non-denaturing PAGE-SDS followed by a Western blot. Data were represented as Pearson correlation coefficient _(__*p* value)_

	Ad_**pl**_ level		HMW form proportion (%)	
Correlation	Non-hypoxemic (*n* = 46)	Hypoxemic (*n* = 54)	Non-hypoxemic (*n* = 46)	Hypoxemic (*n* = 54)
BMI (kg/m^2^)	−0,428* _(*p* = 0,003)_	− 0,422* _(*p* = 0,001)_	− 0,340* _(*p* = 0,021)_	−0,012 _(*p* = 0,934)_
*P*aO_2_ (mmHg)	− 0,161 _(*p* = 0,287)_	0,214 _(*p* = 0,120)_	−0,047 _(*p* = 0,757)_	0,113 _(__*p* = 0,416)_
*P*aCO_2_ (mmHg)	−0,148 _(*p* = 0,328)_	0,074 _(__*p* = 0,600)_	−0,221 _(*p* = 0,141)_	0,163 _(__*p* = 0,247)_
FEV1 (%pv)	−0,033 _(*p* = 0,826)_	− 0,181 _(*p* = 0,190)_	− 0,237 _(*p* = 0,113)_	0,105 _(__*p* = 0,451)_
FEV1/FVC	0,052 _(__*p* = 0,732)_	−0,155 _(__*p* = 0,262)_	−0,080 _(*p* = 0,598)_	0,038 _(__*p* = 0,787)_
TL_CO_ (%pv)	−0,155 _(__*p* = 0,315)_	−0,142 _(*p* = 0,306)_	− 0,314* _(__*p* = 0,043)_	0,263 _(__*p* = 0,055)_
TLC (%pv)	0,038 _(__*p* = 0,811)_	−0,104 _(__*p* = 0,464)_	−0,007 _(__*p* = 0,963)_	− 0,181 _(__*p* = 0,198)_
RV (%pv)	0,043 _(__*p* = 0,786)_	0,003 _(__*p* = 0,983)_	0,166 _(__*p* = 0,294)_	−0,232 _(__*p* = 0,097)_

* Pearson correlation coefficient with a *p* < 0.05

### Impact of gender difference on Ad_pl_ level and HMW forms

**Fig. 2:**
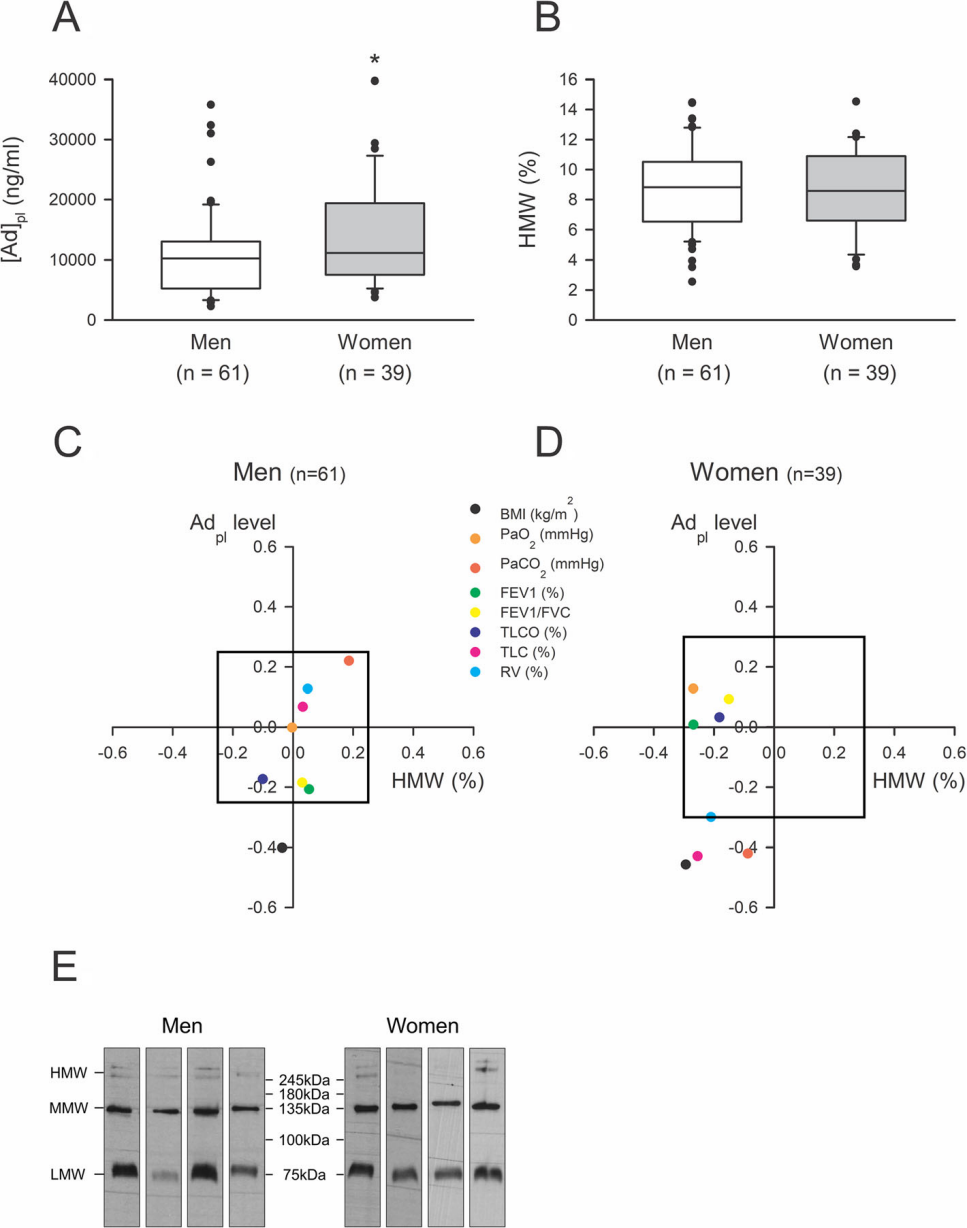
**a-b** Ad_pl_ level (**a**) and HMW form proportion (**b**) in men and women COPD patients. Ad_pl_ level was measured by ELISA and HMW form proportion was determined using a non-denaturing PAGE-SDS followed by a Western blot. Data were represented as boxplots (5th and 95th percentiles). * *p* < 0.05; Rank-Sum test. **c-d** Correlation between Ad_pl_ level (vertical), or HMW form proportion (horizontal) and lung function parameters in men (**a**) and women (**b**) COPD patients. Pearson correlation coefficients (R) between every parameter and either Ad_pl_ level or HMW form proportion were calculated and reported as a point on the graph. The box in the graph represents the critical value for Pearson correlation coefficient to obtain a *p* < 0.05. Therefore, every point outside the box corresponds to a significant correlation. **e** Representative blots of Ad form proportion in men and women, determined by using non-denaturant PAGE-SDS followed by a Western blot. HMW forms correspond to the higher molecular weight bands. Admer/total Ad ratios were obtained after densitometric analysis

**Fig. 3:**
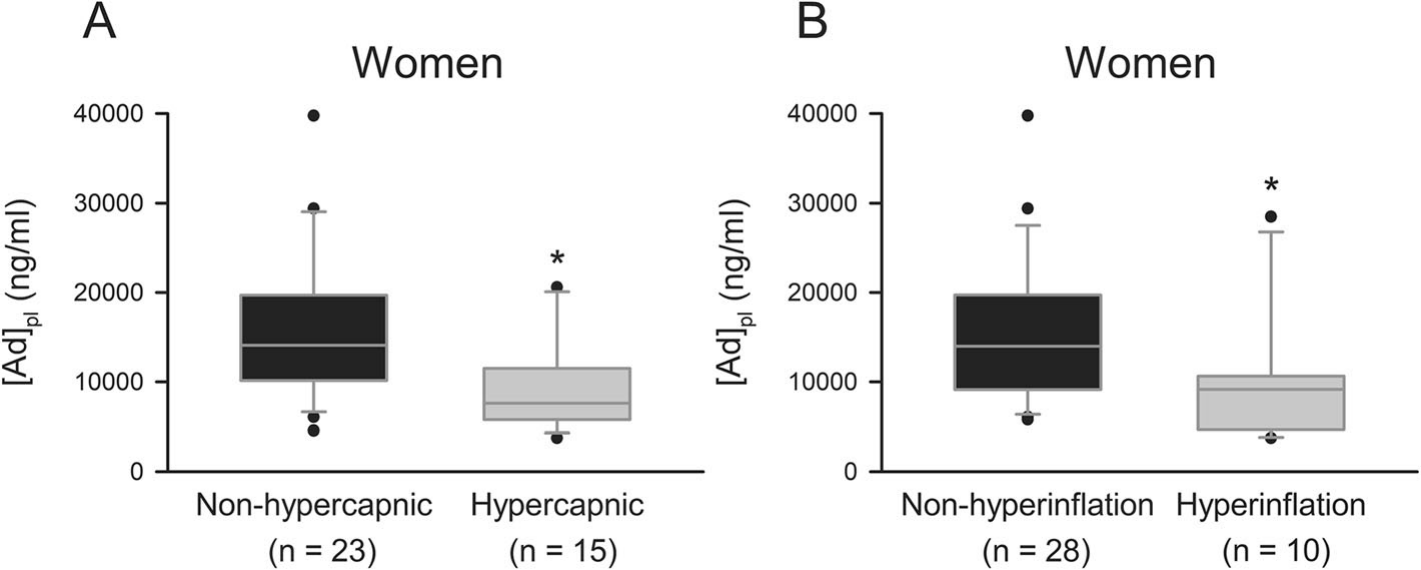
Ad_pl_ level in women with or without hypercapnia (**a**) or lung hyperinflation (**b**). Ad_pl_ level was measured by ELISA. Data were represented as boxplots (5th and 95th percentiles). * *p* < 0.05; Rank-Sum test

As gender differences in total and HMW Ad levels were previously observed in many studies [35, 36], we evaluated these parameters in men and women and correlated these values with lung function parameters. We found a higher Ad_pl_ level in women compared with men, while no difference between HMW form proportions was observed (Fig. 2a-b, e). As previously, in both groups, Ad_pl_ level was negatively correlated with BMI (Fig. 2c-d, Table 3). In women, we observed a significant negative correlation between Ad_pl_ level and TLC, as well as with *P*aCO_2_. These observations were in accordance with the decrease of Ad_pl_ level in hypercapnic women (*P*aCO_2_ > 45 mmHg) compared with normocapnic women (*p* < 0.05) (Fig. 3a). A reduced Ad_pl_ level was also observed in women characterized by hyperinflated lungs (TLC > 115%pv) (Fig. 3b). Concerning HMW form proportion, no gender difference and no correlation with BMI, arterial gas values, or lung parameters were detected.

**Table 3:** Correlation between lung function parameters and Ad_pl_ level or HMW form proportion in men and women COPD patients. Ad_pl_ level was measured by ELISA and HMW form proportion was determined using a non-denaturing PAGE-SDS followed by a Western blot. Data were represented as Pearson correlation coefficient _(*p* value)_

	Ad_**pl**_ level		HMW form proportion (%)	
Correlation	Men (*n* = 61)	Women (*n* = 39)	Men (*n* = 61)	Women (*n* = 39)
BMI (kg/m^2^)	−0,402* _(*p* = 0,001)_	−0,458* _(*p* = 0,003)_	− 0,036 _(*p* = 0,783)_	− 0,293 _(__*p* = 0,070)_
*P*aO_2_ (mmHg)	− 0,002 _(*p* = 0,989)_	0,127 _(*p* = 0,440)_	− 0,003 _(0,984)_	− 0,269 _(*p* = 0,098)_
*P*aCO_2_ (mmHg)	0,220 _(__*p* = 0,0913_)	−0,421* _(*p* = 0,008)_	0,186 _(__*p* = 0,155)_	−0,087 _(*p* = 0,603)_
FEV1 (%pv)	−0,208 _(__*p* = 0,109)_	0,008 _(__*p* = 0,962)_	0,054 _(__*p* = 0,682)_	−0,268 _(__*p* = 0,099)_
FEV1/FVC	−0,185 _(__*p* = 0,153)_	0,093 _(__*p* = 0,575)_	0,031 _(__*p* = 0,814)_	−0,150 _(__*p* = 0,362)_
TL_CO_ (%pv)	−0,174 _(__*p* = 0,181)_	0,032 _(__*p* = 0,851)_	−0,100 _(__*p* = 0,441)_	−0,182 _(__*p* = 0,282)_
TLC (%pv)	0,067 _(__*p* = 0,622)_	−0,429* _(__*p* = 0,007)_	0,033 _(__*p* = 0,811)_	−0,254 _(__*p* = 0,124)_
RV (%pv)	0,127 _(__*p* = 0,352)_	−0,300 _(__*p* = 0,067)_	0,049 _(__*p* = 0,721)_	−0,210 _(__*p* = 0,206)_

* Pearson correlation coefficient with a *p* < 0.05

### Effect of combined gender difference and hypoxia on Ad_pl_ level and HMW forms

**Fig. 4:**
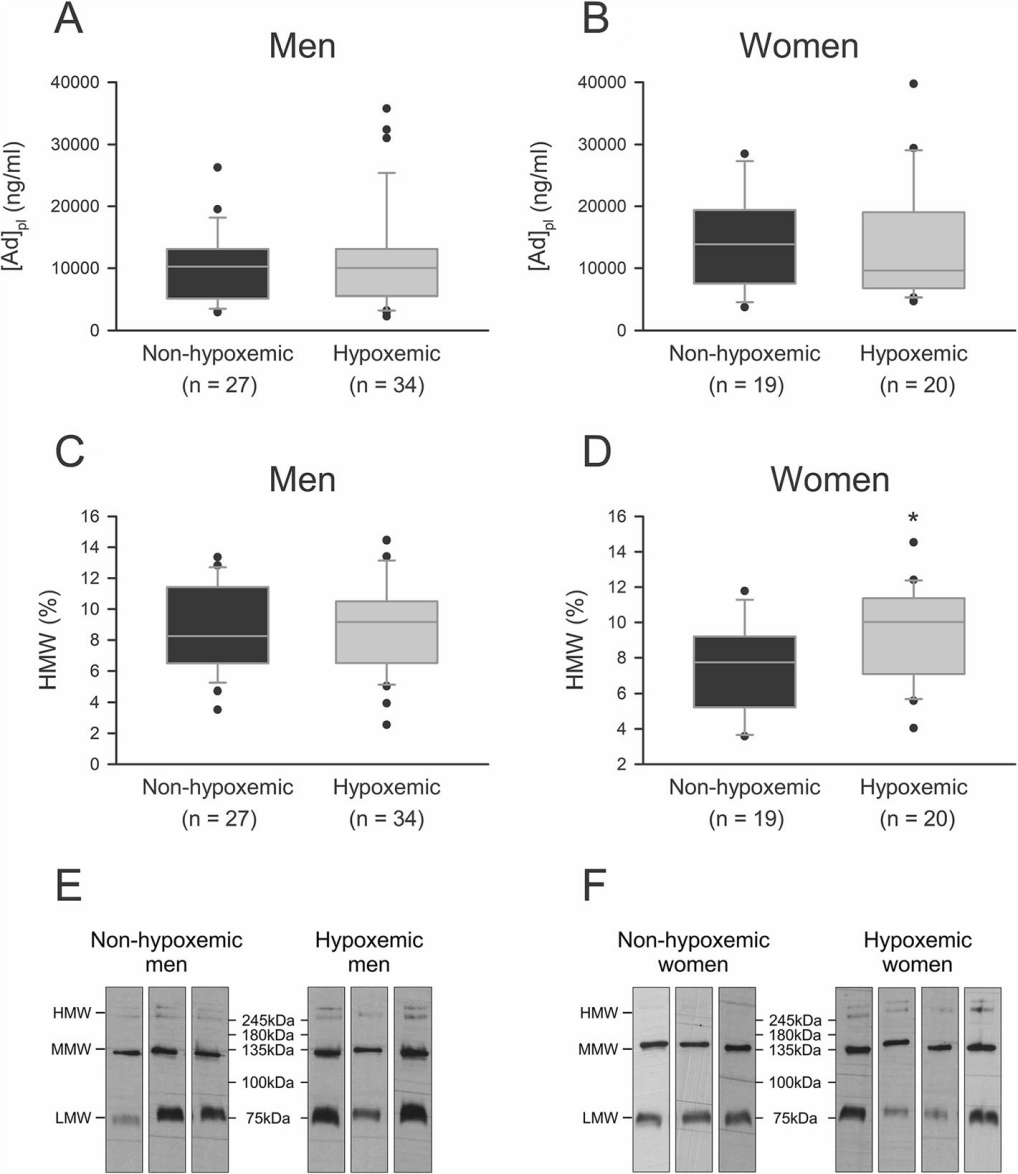
Ad_pl_ level (**a-b**) and HMW form proportion (**c-d**) in hypoxemic and non-hypoxemic men and women COPD patients. Ad_pl_ level was measured by ELISA and HMW form proportion was determined using a non-denaturing PAGE-SDS followed by a Western blot. Data were represented as boxplots (5th and 95th percentiles). * *p* < 0.05; Rank-Sum test. **e-f** Representative blots of Ad form proportion in non-hypoxemic and hypoxemic men (**e**) as well as in non-hypoxemic and hypoxemic women (**f**). HMW form proportion was determined by using non-denaturant PAGE-SDS followed by a Western blot and corresponds to the higher molecular weight bands. Admer/total Ad ratios were obtained after densitometric analysis

**Fig. 5:**
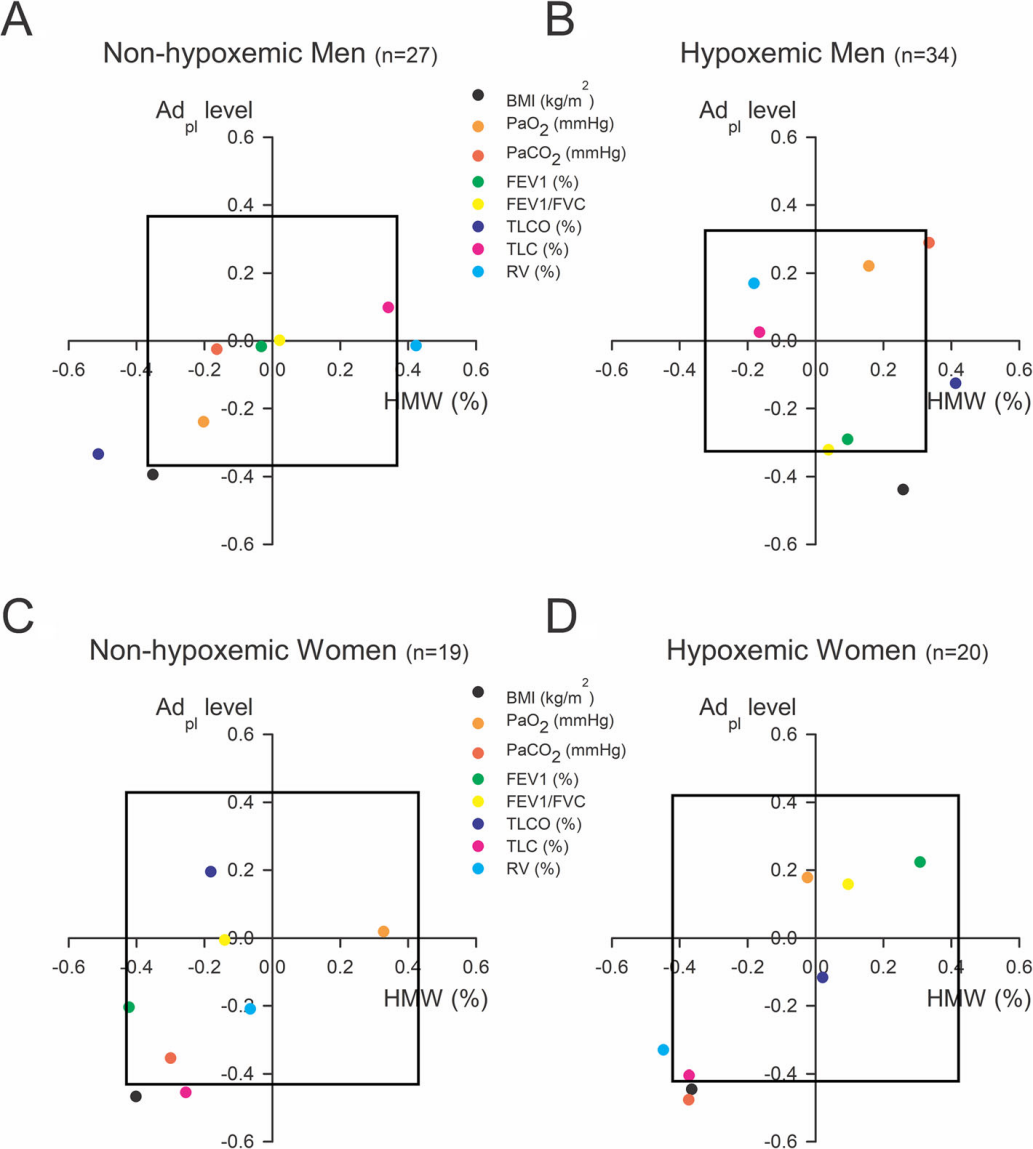
Relationship between Ad_pl_ level, (vertical), HMW form proportion (horizontal) and lung function parameters in non-hypoxemic men (**a**), hypoxemic men (**b**), non-hypoxemic women (**c**) and hypoxemic women (**d**) COPD patients. Ad_pl_ level was measured by ELISA and HMW form proportion was determined using a non-denaturing PAGE-SDS followed by a Western blot. Pearson correlation coefficients (R) between every parameter and either Ad_pl_ level or HMW form proportion were calculated and were represented as a point on the graph. The box in the graph represents the critical value for Pearson correlation coefficient to obtain a *p* < 0.05. Therefore, every point outside the box corresponds to a significant correlation

We did not observe any modification in total or HMW Ad level between hypoxemic and non-hypoxemic men (Fig. 4). In women, hypoxaemia did not modify total Ad_pl_ level but increased HMW form proportion. Indeed, as shown on representative blots (Fig. 4f), the higher bands, corresponding to HMW Ad forms, are more intense in hypoxemic women compared with non-hypoxemic women. As previously, whatever the gender, Ad_pl_ level was negatively correlated with BMI in hypoxemic and non-hypoxemic groups (Fig. 5, Table 4). A negative correlation between total Ad_pl_ level and TLC was statistically significant in non-hypoxemic women, and borderline in hypoxemic women (*p* = 0.07). Ad_pl_ level was also negatively correlated with *P*aCO_2_ in hypoxemic women. Regarding HMW forms, we observed a positive correlation between HMW proportion and RV in non-hypoxemic men (Fig. 5, Table 5). We also detected an opposite relationship between TL_CO_ and HMW level in hypoxemic and non-hypoxemic men. While a positive correlation was observed in hypoxemic men, a negative correlation was detected in non-hypoxemic men. This relationship did not appear in women. Although, we observed that HMW level was negatively associated with RV in hypoxemic women.

**Table 4:** Correlation between lung function parameters and Ad_pl_ level in hypoxemic and non-hypoxemic men and women COPD patients. Ad_pl_ level was measured by ELISA and HMW form proportion was determined using a non-denaturing PAGE-SDS followed by a Western blot. Data were represented as Pearson correlation coefficient _(*p* value)_

	Ad_**pl**_ level		Ad_**pl**_ level	
Correlation	Non-hypoxemic Men (*n* = 27)	Hypoxemic Men (*n* = 34)	Non-hypoxemic Women (*n* = 19)	Hypoxemic Women (*n* = 20)
BMI (kg/m^2^)	−0,395* _(*p* = 0,042)_	−0,439* _(*p* = 0,009)_	− 0,467* _(*p* = 0,044)_	− 0,446* _(_*p* _= 0,049)_
*P*aO_2_ (mmHg)	− 0,239 _(__*p* = 0,231)_	0,221 _(__*p* = 0,210)_	0,019 _(__*p* = 0,939)_	0,177 _(__*p* = 0,454)_
*P*aCO_2_ (mmHg)	−0,025 _(__*p* = 0,901)_	0,288 _(__*p* = 0,104)_	−0,355 _(__*p* = 0,136)_	−0,477* _(__*p* = 0,039)_
FEV1 (%pv)	−0,017 _(__*p* = 0,932)_	−0,290 _(__*p* = 0,096)_	− 0,204 _(__*p* = 0,401)_	0,223 _(__*p* = 0,344)_
FEV1/FVC	0,001 _(__*p* = 0,995)_	−0,321 _(__*p* = 0,064)_	−0,006 _(__*p* = 0,981)_	0,158 _(__*p* = 0,506)_
TL_CO_ (%pv)	−0,334 _(0,095)_	−0,126 _(__*p* = 0,478)_	0,195 _(__*p* = 0,454)_	−0,116 _(__*p* = 0,625)_
TLC (%pv)	0,098 _(__*p* = 0,647)_	0,025 _(__*p* = 0,892)_	−0,455* _(__*p* = 0,05)_	−0,405 _(__*p* = 0,076)_
RV (%pv)	−0,014 _(__*p* = 0,947)_	0,169 _(__*p* = 0,354)_	−0,209 _(__*p* = 0,404)_	− 0,331 _(__*p* = 0,155)_

* Pearson correlation coefficient with a *p* < 0.05

**Table 5:** Correlation between lung function parameters and HMW form proportion in hypoxemic and non-hypoxemic men and women COPD patients. Ad_pl_ level was measured by ELISA and HMW form proportion was determined using a non-denaturing PAGE-SDS followed by a Western blot. Data were represented as Pearson correlation coefficient _(__*p* value)_

	HMW form proportion (%)		HMW form proportion (%)	
Correlation	Non-hypoxemic Men (*n* = 27)	Hypoxemic Men (*n* = 34)	Non-hypoxemic Women (*n* = 19)	Hypoxemic Women (*n* = 20)
BMI (kg/m^2^)	−0,352 _(__*p* = 0,072)_	0,258 _(__*p* = 0,141)_	−0,401 _(*p* = 0,088)_	− 0,365 _(*p* = 0,114)_
*P*aO_2_ (mmHg)	−0,202 _(__*p* = 0,311)_	0,157 _(__*p* = 0,374)_	0,328 _(__*p* = 0,170)_	−0,023 _(__*p* = 0,922)_
*P*aCO_2_ (mmHg)	−0,163 _(__*p* = 0,416)_	0,335 _(__*p* = 0,057)_	−0,299 _(__*p* = 0,213)_	−0,373 _(__*p* = 0,116)_
FEV1 (%pv)	−0,032 _(__*p* = 0,874)_	0,095 _(__*p* = 0,593)_	−0,422 _(__*p* = 0,072)_	0,308 _(__*p* = 0,186)_
FEV1/FVC	0,022 _(__*p* = 0,915)_	0,039 _(__*p* = 0,829)_	−0,140 _(__*p* = 0,568)_	0,096 _(__*p* = 0,686)_
TL_CO_ (%pv)	−0,513* _(__*p* = 0,007)_	0,413* _(__*p* = 0,015)_	−0,181 _(__*p* = 0,487)_	0,021 _(__*p* = 0,930)_
TLC (%pv)	0,341 _(__*p* = 0,102)_	−0,165 _(__*p* = 0,367)_	−0,255 _(__*p* = 0,308)_	− 0,372 _(__*p* = 0,106)_
RV (%pv)	0,423* _(__*p* = 0,039)_	−0,181 _(__*p* = 0,321)_	−0,065 _(__*p* = 0,798)_	− 0,448* _(__*p* = 0,047)_

* Pearson correlation coefficient with a *p* < 0.05

## Discussion

We postulated that gender and hypoxaemia could influence Ad_pl_ level as well as its multimers and therefore contribute to the appearance of a distinct endotype associated to an altered CVD risk. Our results showed a gender difference in Ad_pl_ level. Women exhibited a higher Ad_pl_ level compared with men. While this discrepancy was previously described [37, 38], the mechanism underlying this divergence is still investigated. Testosterone level [39–42] and the difference in adipose tissue distribution between men and women were previously mentioned [43]. Other mechanisms could also explain this gender variation in Ad_pl_ level in COPD patients. In our study, differences in men and women Ad_pl_ level were associated with divergent clinical characteristics, among which the increased TCL and RV %pv in women, reflecting hyperinflation and air-trapping respectively.

The increased TLC %pv was correlated with a reduced Ad_pl_ level. As pro-inflammatory cytokines were previously demonstrated to reduce Ad expression in adipocytes [44], it could be hypothesised that inflammatory state in these patients modulates Ad_pl_ level. Indeed, Rubinsztajn et al. observed that patients with hyperinflation had elevated inflammatory markers [21]. Another explanation supporting this hypothesis could be the gender difference in inflammatory processes [45–47]. The exact mechanism remains unclear. Rathod et al. showed that a divergent hormonal status could be involved in this regulation [46], whereas the study of Casimir et al. observed that some genes on X chromosome, involved in the inflammatory pathway, were overexpressed in women [45]. While this divergence in the inflammatory state was not previously described in COPD, this phenomenon is well known in patients with asthma, with a more pronounced inflammatory response within the airway wall in women [48–50]. Therefore, more studies are necessary for elucidating the potential role of inflammation in the association between Ad_pl_ level and TLC %pv in women with COPD.

Moreover, Ad_pl_ level in women was negatively associated with *P*aCO_2_, especially in the hypoxemic subgroup. These observations are consistent with previous studies that found a decreased Ad_pl_ level and a higher *P*aCO_2_ in mice with acute lung injury [51] or in patients with hypoventilation syndrome [52]. Moreover, Dimoulis et al. observed that Ad_pl_ level was negatively correlated with bicarbonate level [53]. They also found that, in stable hypercapnic COPD, non-invasive ventilation reduced *P*aCO_2_ and increased *P*aO_2_ and Ad_pl_ level from the first month of intervention, without any change in BMI [54]. Therefore, our study suggested that functional alterations such as hyperinflation, air trapping and impaired gas exchange also modulated Ad_pl_ level in COPD patients.

In addition to its potential modulation of Ad_pl_ level through its association with hypercapnia, hypoxaemia was associated to an increased level of HMW forms in women. Interestingly, these observations were in accordance with our previous study in which an increased HMW form proportion in a murine model of hypoxaemia was observed [34]. In this model, the only difference between the active and control groups was the exposure to hypoxia. However, we also observed that the increased HMW form proportion was associated to a decreased AdipoR protein level in different tissues [55, 56]. Further studies are therefore needed to better understand the impact of an increased level of HMW forms in hypoxemic women. No modulation of Ad_pl_ level or HMW form proportion were observed among hypoxemic and non-hypoxemic men, suggesting that Ad is modulated by different mechanisms in men and women. One explanation could be the presence of a gender-difference in the inflammatory response, as previously mentioned. Indeed, in different pathological contexts, previous studies reported that women were affected to a more extended level by a pro-inflammatory state but respond more vigorously [57–59].

Altogether, our results suggested that gender difference in Ad_pl_ level observed in our COPD patients could be explained by a divergent pathophysiology in men and women. Indeed, we observed a gender divergence in TLC and RV, without any difference in BMI. The increase of TLC and RV %pv observed in women in our study was also found in other recent studies [60–62]. These data suggested that, for a given FEV1, women had a more pronounced air trapping or hyperinflation than men, while hyperinflation was quite limited in our study (mean TLC was 105%pv in women and 87%pv in men). These observations are consistent with Grabicki et al. that showed that COPD women had more hyperinflation, air trapping and comorbidities, inducing a higher risk of mortality [61]. In addition, in women, this increased air trapping also led to the development of hypercapnia [63]. Our results also contrasted with previous studies in which men exhibited more emphysema than women. By using CT scan, previous studies observed that the percentage of low attenuation area was higher in men than in women [64–66]. However, the study of Hardin et al. [67] highlighted that gender difference in CT-determined emphysema is dependent on the severity of airway obstruction (GOLD classification). Despite of these considerations, it is interesting to note that the difference in radiological emphysema did not appear in functional hyperinflation in Martinez’s study. These results underlined the contrast between radiological evaluations in favour of more emphysema in men, and functional measures reflecting probably a higher air trapping in women. This study has some limitations. As we evaluated the effect of the hypoxaemia component in COPD patient, we separated our cohort according to the *P*aO_2_ (≤55 mmHg). However, patients in such severe condition received LTOT in order to reduce the risk of complications and mortality. It was therefore difficult to tolerate a washing period from oxygen therapy for those already on this treatment at the inclusion time. Treatment-naive patients should be selected and modulation of Ad_pl_ level and HMW form proportion could then be studied before and after LTOT in the same patient. In our study, the main confounding factors (age, BMI, gender) affecting Ad_pl_ level in COPD patients [68], were taken into consideration. However, we cannot exclude any influence of other concomitant factors such as medication, physical activity or cardio-vascular co-morbidities.

## Conclusions

Altogether, these data suggested that men and women with severe hypoxaemia exhibited a different pathophysiology of COPD, which is linked to Ad modulation. Indeed, an increased Ad_pl_ level was observed in COPD women and was associated with a distinct pattern of functional alterations (for the same age, BMI or obstruction severity): women had more hyperinflation, air-trapping and hypercapnia, which, in association with hypoxaemia, could contribute to a modulation of Ad_pl_ level. These variations were accompanied with an increased level of HMW forms in hypoxemic women. All these results suggest the development of a distinct endotypic presentation, based on gender, with a more pronounced bronchiolar damages possibly associated with an inflammatory state, leading to an increased air-trapping. Consequently, a long-term study should be realized to evaluate if these modulations of total Ad_pl_ and HMW forms are associated with a better survival or with a lower risk of comorbidities.

## Supplementary information

**Additional file 1.**

**Additional file 2.**

## Data Availability

The datasets supporting the conclusion of this article are included within the article and its additional file.

## Abbreviations

Ad: Adiponectin
Ad_pl_: Ad plasmatic
COPD: Chronic obstructive pulmonary disease
CVD: Cardiovascular diseases
FEV1: Forced expiratory volume in one second
FVC: Forced vital capacity
HMW: High molecular weight
IC: Inspiratory capacity
LMW: Low molecular weight
MMW: Medium molecular weight
*P*aO_2_: Arterial oxygen partial pressure
*P*aCO_2_: Carbon dioxide partial pressure
R: Pearson correlation coefficient
RV: Residual volume
TLC: Total lung capacity
TLco: Carbon monoxide transfer factor
%pv: Percentage of the predicted value
