# Supplementary material for: Chronic hypoxaemia and gender status modulate adiponectin plasmatic level and its multimer proportion in severe COPD patients: new endotypic presentation?
Source: BMC Pulm Med. 2020 Oct 1;20:255. doi: 10.1186/s12890-020-01288-3 (PMC7528580; doi:10.1186/s12890-020-01288-3)
Supplement: Supplementary file 1 — Additional file 1. [file 12890_2020_1288_MOESM1_ESM.docx]

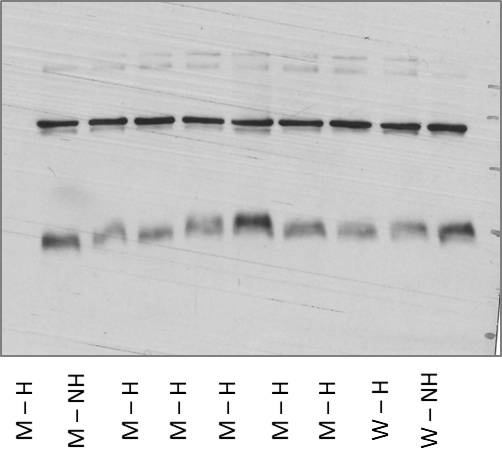


Figure SI 1: Representative blots of Ad form proportion in COPD men or women that were hypoxemic or not, determined by using non-denaturant PAGE-SDS followed by a Western blot. HMW forms correspond to the higher molecular weight bands. Admer/total Ad ratios were obtained after densitometric analysis. M-H: Men-Hypoxemic; M-NH: Men-Non-hypoxemic; W: Women-Hypoxemic; W-NH: Women-Non-Hypoxemic.


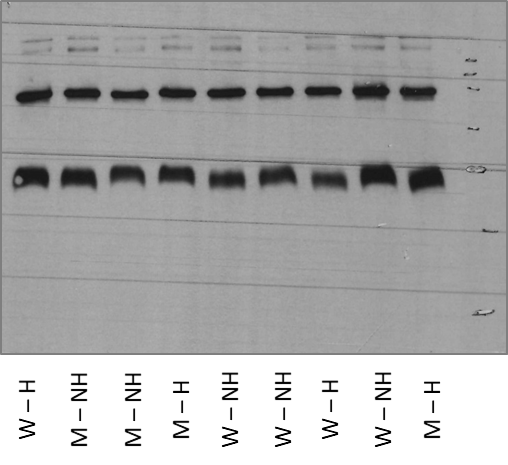


Figure SI 2: Representative blots of Ad form proportion in COPD men or women that were hypoxemic or not, determined by using non-denaturant PAGE-SDS followed by a Western blot. HMW forms correspond to the higher molecular weight bands. Admer/total Ad ratios were obtained after densitometric analysis. M-H: Men-Hypoxemic; M-NH: Men-Non-hypoxemic; W: Women-Hypoxemic; W-NH: Women-Non-Hypoxemic.


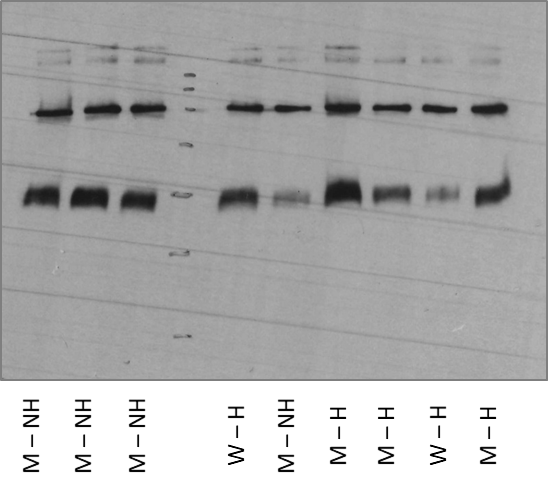


Figure SI 3: Representative blots of Ad form proportion in COPD men or women that were hypoxemic or not, determined by using non-denaturant PAGE-SDS followed by a Western blot. HMW forms correspond to the higher molecular weight bands. Admer/total Ad ratios were obtained after densitometric analysis. M-H: Men-Hypoxemic; M-NH: Men-Non-hypoxemic; W: Women-Hypoxemic; W-NH: Women-Non-Hypoxemic.


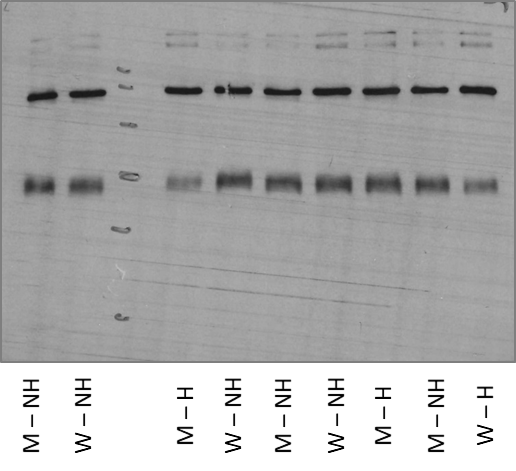


Figure SI 4: Representative blots of Ad form proportion in COPD men or women that were hypoxemic or not, determined by using non-denaturant PAGE-SDS followed by a Western blot. HMW forms correspond to the higher molecular weight bands. Admer/total Ad ratios were obtained after densitometric analysis. M-H: Men-Hypoxemic; M-NH: Men-Non-hypoxemic; W: Women-Hypoxemic; W-NH: Women-Non-Hypoxemic.


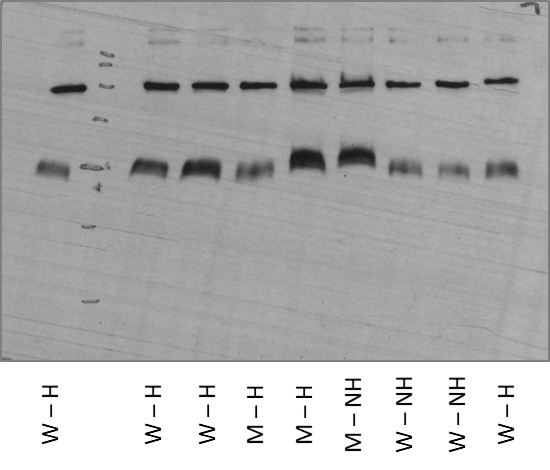


Figure SI 5: Representative blots of Ad form proportion in COPD men or women that were hypoxemic or not, determined by using non-denaturant PAGE-SDS followed by a Western blot. HMW forms correspond to the higher molecular weight bands. Admer/total Ad ratios were obtained after densitometric analysis. M-H: Men-Hypoxemic; M-NH: Men-Non-hypoxemic; W: Women-Hypoxemic; W-NH: Women-Non-Hypoxemic.


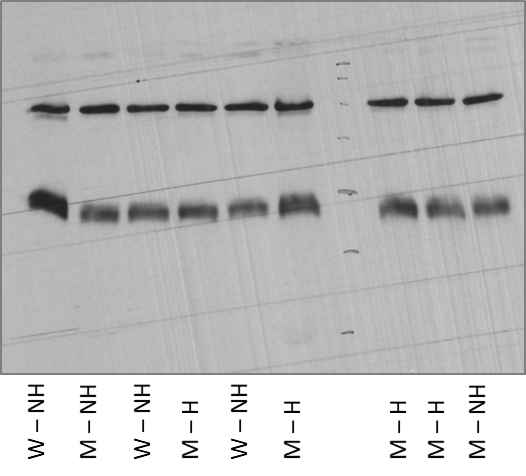


Figure SI 6: Representative blots of Ad form proportion in COPD men or women that were hypoxemic or not, determined by using non-denaturant PAGE-SDS followed by a Western blot. HMW forms correspond to the higher molecular weight bands. Admer/total Ad ratios were obtained after densitometric analysis. M-H: Men-Hypoxemic; M-NH: Men-Non-hypoxemic; W: Women-Hypoxemic; W-NH: Women-Non-Hypoxemic.


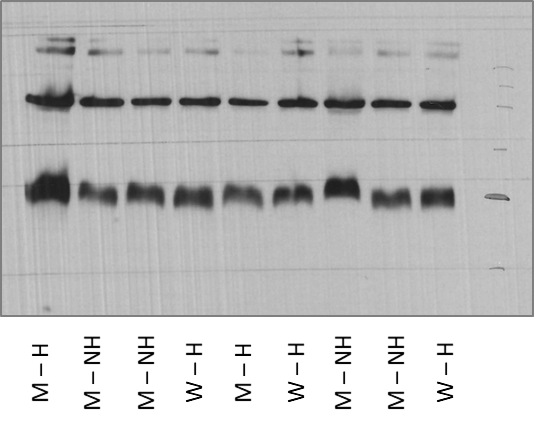


Figure SI 7: Representative blots of Ad form proportion in COPD men or women that were hypoxemic or not, determined by using non-denaturant PAGE-SDS followed by a Western blot. HMW forms correspond to the higher molecular weight bands. Admer/total Ad ratios were obtained after densitometric analysis. M-H: Men-Hypoxemic; M-NH: Men-Non-hypoxemic; W: Women-Hypoxemic; W-NH: Women-Non-Hypoxemic.


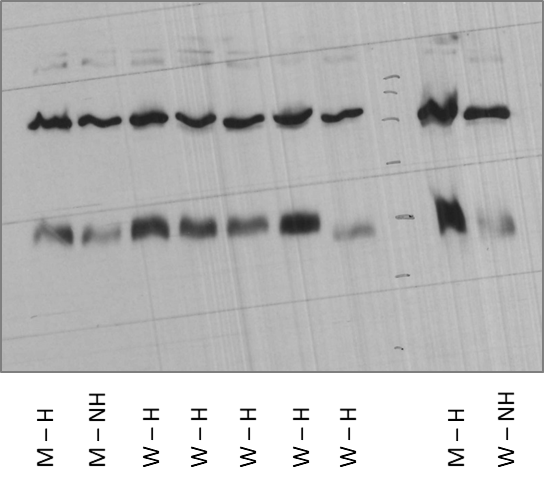


Figure SI 8: Representative blots of Ad form proportion in COPD men or women that were hypoxemic or not, determined by using non-denaturant PAGE-SDS followed by a Western blot. HMW forms correspond to the higher molecular weight bands. Admer/total Ad ratios were obtained after densitometric analysis. M-H: Men-Hypoxemic; M-NH: Men-Non-hypoxemic; W: Women-Hypoxemic; W-NH: Women-Non-Hypoxemic.


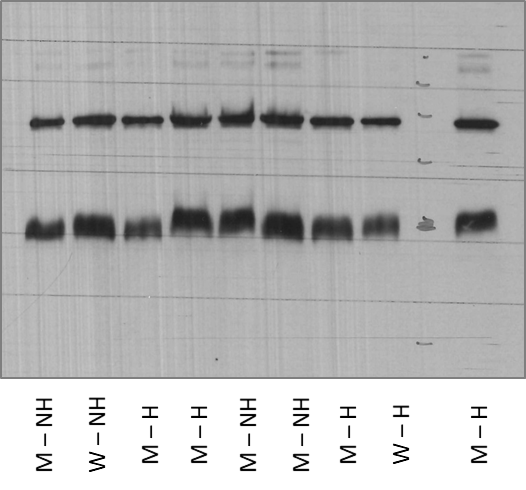


Figure SI 9: Representative blots of Ad form proportion in COPD men or women that were hypoxemic or not, determined by using non-denaturant PAGE-SDS followed by a Western blot. HMW forms correspond to the higher molecular weight bands. Admer/total Ad ratios were obtained after densitometric analysis. M-H: Men-Hypoxemic; M-NH: Men-Non-hypoxemic; W: Women-Hypoxemic; W-NH: Women-Non-Hypoxemic.


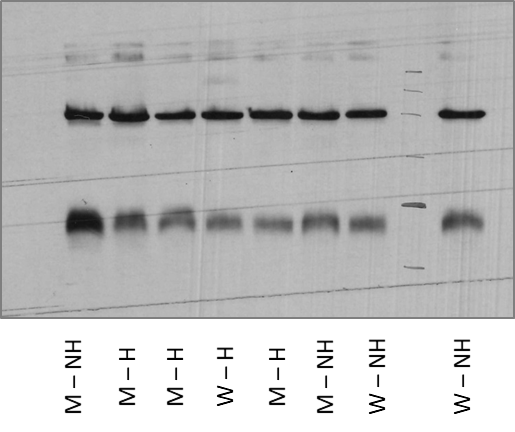


Figure SI 10: Representative blots of Ad form proportion in COPD men or women that were hypoxemic or not, determined by using non-denaturant PAGE-SDS followed by a Western blot. HMW forms correspond to the higher molecular weight bands. Admer/total Ad ratios were obtained after densitometric analysis. M-H: Men-Hypoxemic; M-NH: Men-Non-hypoxemic; W: Women-Hypoxemic; W-NH: Women-Non-Hypoxemic.


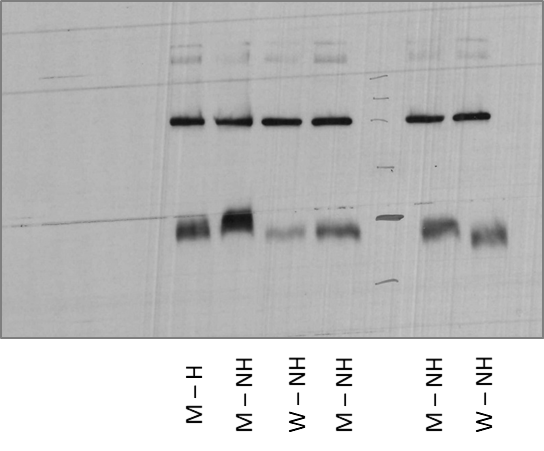


Figure SI 11: Representative blots of Ad form proportion in COPD men or women that were hypoxemic or not, determined by using non-denaturant PAGE-SDS followed by a Western blot. HMW forms correspond to the higher molecular weight bands. Admer/total Ad ratios were obtained after densitometric analysis. M-H: Men-Hypoxemic; M-NH: Men-Non-hypoxemic; W: Women-Hypoxemic; W-NH: Women-Non-Hypoxemic.


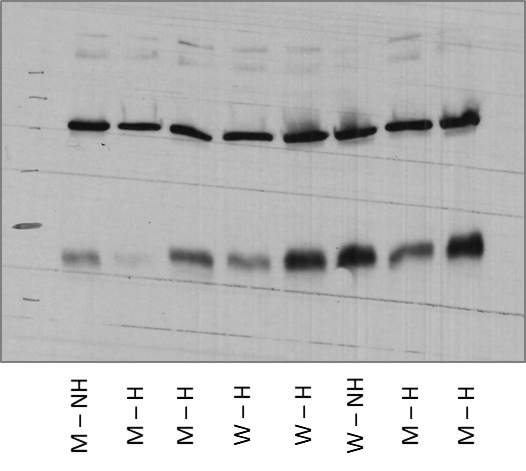


Figure SI 12: Representative blots of Ad form proportion in COPD men or women that were hypoxemic or not, determined by using non-denaturant PAGE-SDS followed by a Western blot. HMW forms correspond to the higher molecular weight bands. Admer/total Ad ratios were obtained after densitometric analysis. M-H: Men-Hypoxemic; M-NH: Men-Non-hypoxemic; W: Women-Hypoxemic; W-NH: Women-Non-Hypoxemic.
